# Personalized federated learning for abdominal multi‐organ segmentation based on frequency domain aggregation

**DOI:** 10.1002/acm2.14602

**Published:** 2024-12-05

**Authors:** Hao Fu, Jian Zhang, Lanlan Chen, Junzhong Zou

**Affiliations:** ^1^ Department of Automation, School of Information Science and Engineering East China University of Science and Technology Shanghai China

**Keywords:** abdominal multi‐organ segmentation, federated learning, medical image, personalized federated learning

## Abstract

**Purpose:**

The training of deep learning (DL) models in medical images requires large amounts of sensitive patient data. However, acquiring adequately labeled datasets is challenging because of the heavy workload of manual annotations and the stringent privacy protocols.

**Methods:**

Federated learning (FL) provides an alternative approach in which a coalition of clients collaboratively trains models without exchanging the underlying datasets. In this study, a novel Personalized Federated Learning Framework (PAF‐Fed) is presented for abdominal multi‐organ segmentation. Different from traditional FL algorithms, PAF‐Fed selectively gathers partial model parameters for inter‐client collaboration, retaining the remaining parameters to learn local data distributions at individual sites. Additionally, the Fourier Transform with the Self‐attention mechanism is employed to aggregate the low‐frequency components of parameters, promoting the extraction of shared knowledge and tackling statistical heterogeneity from diverse client datasets.

**Results:**

The proposed method was evaluated on the Combined Healthy Abdominal Organ Segmentation magnetic resonance imaging (MRI) dataset (CHAOS 2019) and a private computed tomography (CT) dataset, achieving an average Dice Similarity Coefficient (DSC) of 72.65% for CHAOS and 85.50% for the private CT dataset, respectively.

**Conclusion:**

The experimental results demonstrate the superiority of our PAF‐Fed by outperforming state‐of‐the‐art FL methods.

## 1 INTRODUCTION

Medical imaging stands as a cornerstone in modern medicine due to its non‐invasion and remarkable efficacy. Nonetheless, conventional medical image analysis in clinical applications predominantly relies on manual observation, which is labor‐intensive, time‐consuming, and prone to obvious inter‐observer variability.^1^ The evolution of artificial intelligence (AI) provides computer‐aided solutions for accelerated medical image analysis and prompt anomaly identification. In recent years, machine learning (ML) and deep learning (DL) have been increasingly applied to medical image analysis, such as anomaly detection, disease classification, lesion segmentation, and computer‐aided diagnosis.^2^ Notably, the automated segmentation of organs has received much attention. This innovative technology plays a vital role in disease diagnosis, treatment guidance, and tumor monitoring, offering invaluable support to radiologists in delineating regions of interest.^3^

**FIGURE 1:**
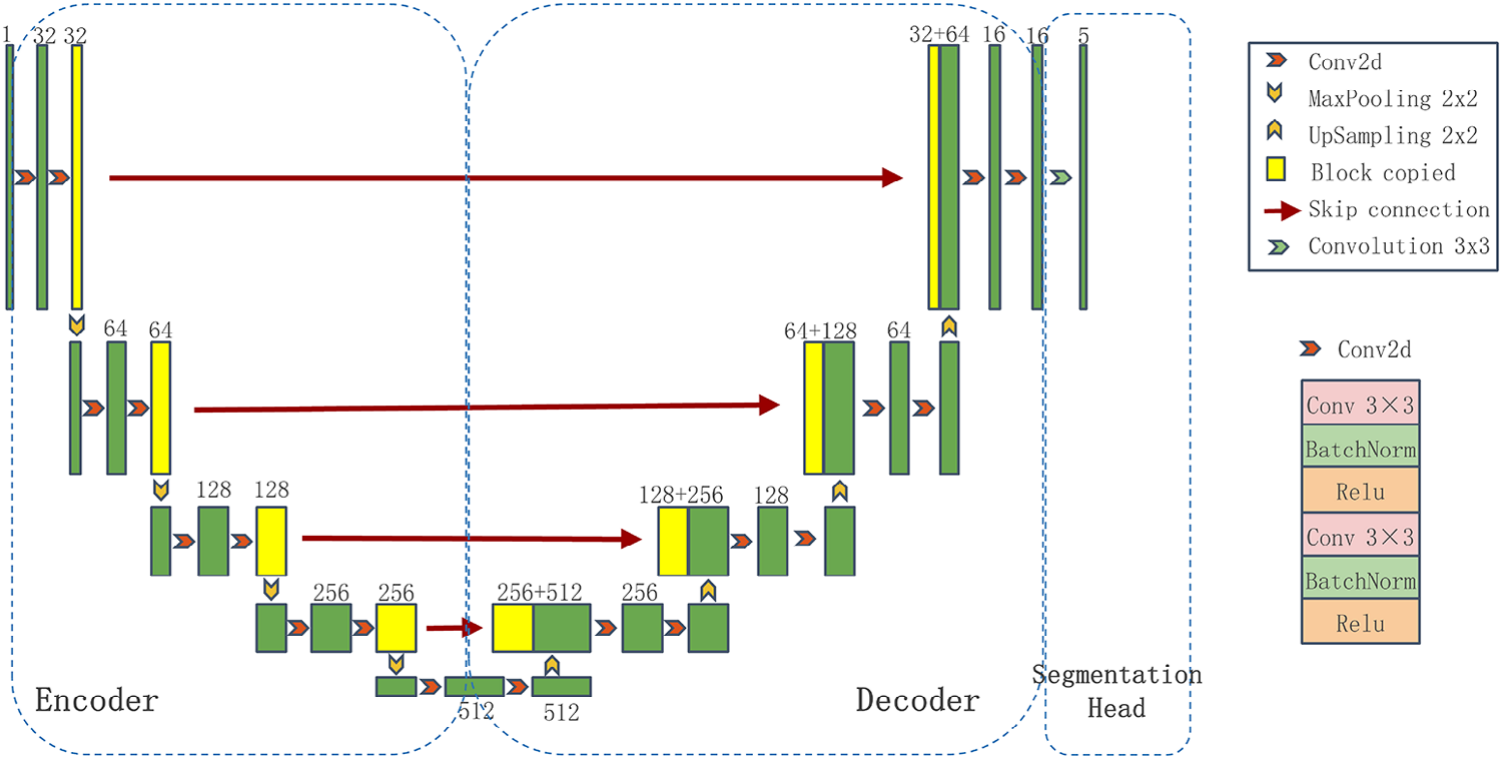
The architecture of U‐Net.

Recently, DL models have achieved outstanding performance in medical imaging tasks. Instead of using handcrafted features, DL models automatically extract features from datasets. For medical imaging segmentation, U‐Net is one of the most popular end‐to‐end fully conventional networks.^4^ As demonstrated in Figure 1, the U‐Net can be divided into an encoder, a decoder, and a segmentation head. Moreover, U‐Net utilizes skip connections from down‐sampling layers to up‐sampling layers to preserve high‐resolution image information. Ambesange et al.^5^ proposed a modified U‐Net for lung segmentation. Czipczer et al.^6^ trained an adaptable model called RP‐UNet for liver segmentation based on CT images. Huang et al.^7^ implemented a full‐scale connected U‐Net for the segmentation of the liver and spleen in abdominal CT scans. Although considerable work has been done, DL networks for abdominal organ segmentation are mainly applied in the following cases^8^: single organ (liver most often), single modality (usually CT), and small private datasets. Consequently, these automated segmentation methods are difficult to meet the requirements of broader clinical generalizability, particularly across diverse MRI structural modalities. Moreover, the training of deep‐learning‐based architectures requires extensive medical images and associated clinical data, exacerbating the difficulty of acquiring sufficiently annotated datasets manually.

**FIGURE 2:**
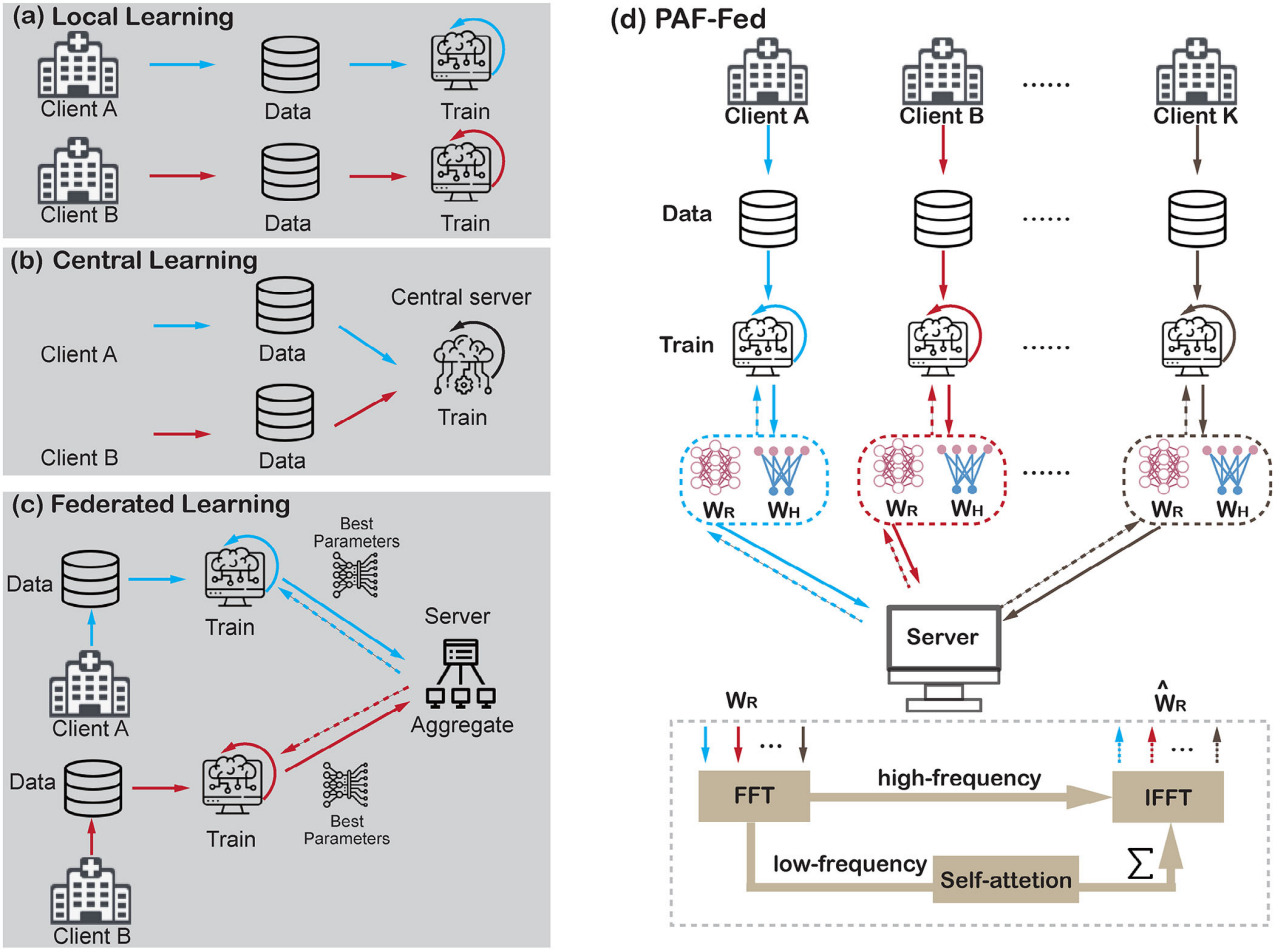
Local, Central, Federated Learning and our proposed algorithm (PAF‐Fed). (a) Local learning (b) Central learning (c) Federated learning (d) PAF‐Fed (Ours). PAF‐FED, Personalized Federated Learning Framework.

Furthermore, deep‐learning models are typically trained using private databases from singular populations, which can lead to poor generalization for practical application^9^ (Figure 2a). Recent years have witnessed the emergence of publicly available medical image datasets for centralized model training (as shown in Figure 2b), such as The Cancer Imaging Archive (TCIA)^10^ and Grand Challenge.^11^ These collaborative efforts are limited by stringent data protection mandates.^12^ Both the European General Data Protection Regulation (GDPR)^13^ and the United States Health Insurance Portability and Accountability Act (HIPAA)^14^ mandate rigorous rules regarding the storage and exchange of personally identifiable data, sparking considerations on data handling, ownership, and AI governance paradigms.

To solve these problems, federated learning (FL) emerges as a game changer for deep‐learning‐based medical image analysis. As illustrated in Figure 2c, FL is a learning paradigm of data governance and privacy by training models collaboratively without direct dataset exchange.^15^ Although originally designed for decentralized training across edge devices and smartphones, FL has caught considerable attention within medical image analysis.^16^ This approach benefits from shared knowledge from each client and avoids the dissemination of sensitive data among disparate clients, thereby overcoming inherent privacy constraints and promoting the development of more generalized models.

Although FL has made promising progress in medical image analysis, the majority of extant studies predominantly fell into the FedAvg algorithm.^17^ This algorithm aggregates global knowledge across diverse clients by directly element‐wise averaging local model parameters.^18^ However, the personalized knowledge learned from local models would be lost in this way. To address this limitation, personalized federated learning (PFL) has emerged as a refined methodology, facilitating enhanced model quality at the client level. Currently, mainstream PFL methods treat the network architecture as two parts, that is, shared and personalized layers. During training, the shared layers are aggregated by FedAvg at the centralized server while the personalized layers are trained on the local client. FedBN regarded the batch normalization (BN) layers as the personalized layers,^19^ on this basis, Andreux et al. proposed SiloBN,^20^ which introduced local‐statistic BN into personalized layers. FedRep^21^ and LG‐FedAvg^22^ selected some of the convolution layers as personalized layers. Besides, some literature used regularization methods for global server aggregation. FedProx addressed the statistical heterogeneity by using L2 regularization during local training.^23^ Nevertheless, most previous works on PFL have been focused on classification tasks, for classification models contain much fewer and simpler layers than the segmentation models. In contrast, the PFL algorithms for image segmentation remain relatively nascent.

In this study, a novel Personalized Federated Learning Framework (PAF‐Fed) for abdominal multi‐organ segmentation is proposed. PAF‐Fed selectively transmits a subset of model parameters to the global server while retaining residual parameters to learn the distinct data characteristics of local clients. The parameter frequency characteristics play pivotal roles in network capability, where low‐frequency components underpin generalizable capabilities and high‐frequency components contain domain‐specific insights.^24^ Furthermore, the Fast Fourier Transform (FFT) and inverse Fast Fourier Transform (IFFT) are harnessed to aggregate low‐frequency parameter components at the global server. Additionally, the spirit of Self‐attention is explored to assign aggregation weight automatically on each client. Finally, quantitative experimental analysis has been conducted on the multi‐sequences MRI (T1‐DUAL, T2‐SPIR) public dataset and our private CT dataset.

In short, this research contributes to the following aspects:

1. This study presents an innovative PFL architecture explicitly designed for abdominal multi‐organ segmentation.
2. A PFL algorithm is designed to distill shared features across clients while preserving unique client‐specific attributes.
3. A new federated weights aggregation mechanism based on FFT is introduced to average the low‐frequency components while retaining the individual high‐frequency components.
4. The Self‐attention mechanism is employed to assign weight automatically to low‐frequency component aggregation.

## 2 METHODS

### 2.1 Overview

In this study, we propose a novel PAF‐Fed for abdominal multi‐organ segmentation, as shown in Figure 2d. Additionally, we summarize the PAF‐Fed method in Algorithm 1. The federation comprises *K* distinct clients participating in the training process. The procedure steps are as follows:

1. Local model training

As for the *k*‐th client, the local model undergoes training utilizing its proprietary dataset. Following *E* epochs of iterative training, the client‐specific weight parameters, denoted as *W_K_*, are obtained.

2. Weight parameter decomposition

The weight parameters *W_K_* are divided into two constituents: a foundational body *W_R_*, emphasizing representation learning, and a personalized component *W_H_* for individual features learning of each local client.

3. Global server model aggregation

The weight parameters $\hat{W_{R}}$ of the global server model are derived after aggregating the low‐frequency components of *W_R_* while retaining the high‐frequency ones. Upon computation, these averaged parameters are delivered back to the corresponding client, thereby instigating an iterative update process for the local model $\hat{W_{K}}$.

This cyclical process is iteratively executed until the global training stage is completed.

ALGORITHM 1 **The pipeline of the PAF‐Fed framework.**

**Table:** 

**Input**: Total number of clients K, global server communication rounds T, Local epochs E,	
B is the local minibatch size, η is the learning rate;	
**Output**: The trained personalized local models ${\left\{W_{k}\right\}}_{k=1}^{K}$	
**Server executes**:	
1.	Initialize: model parameters $W_{0}$, communication rounds t = 0;
2.	Send model parameters $W_{0}$ to each client;
3.	**for** each round in T **do**
4.	**for** each client k in parallel **do**
5.	$W_{Rk}$← ClientUpdate (k, $W_{k}$);
**6**.	**end**
7.	Generate the aggregated model parameter $\hat{W_{R}}$ with Fourier Transform Attention aggregation (FTA);
8.	$\left[\hat{W_{R1}},\hat{W_{R2}},\hat{W_{R3}},\text{…},\hat{W_{Rk}}\right]=\hat{W_{R}}$;
9.	Send $\hat{W_{Rk}}$ back to corresponding client.
**10**.	**end**
**ClientUpdate (**k, $W_{k}$):	
1.	**if** communication rounds t = 0: $W_{k}$ = $W_{0}$
2.	**else**: $W_{k}=\left[W_{Hk}^{E},\hat{W_{Rk}}\right]$
3.	**for** each local epoch *i* from *1* to *E* **do**
4.	**for** batch b∈B **do**
5.	$W_{k}\leftarrow W_{k}-\eta \nabla \ell \left(W_{k};b\right)$;
**6**.	**end**
7.	Compute the global representation parameters $W_{Rk}$;
8.	Return $W_{Rk}$ to server.

### 2.2 Personalized federated learning on BN and prediction layers

The standard form of FL with k clients is

(1) $$\min_{\left(w_{1},\text{…},w_{k}\right)\in R^{d}}\frac{1}{K}\sum_{i=1}^{k}f_{i}\left(w_{i}\right)$$

where *f_i_* is the error function, and *w_i_* is the model parameters of the *i*‐th client. $R^{d}$ is the model parameter sets of k models.

FedAvg learning aims to train a single shared model $\left(w_{1}=w_{2}=w_{3}=\cdots =w_{k}\right)$ that performs well on averaging overall clients. However, the issue of data heterogeneity cannot be ignored, since the data distribution of clients is substantially different from each other. If every client learns a single shared weight parameter by minimizing average loss, the resulting model could perform poorly for many of the clients.

In this study, we aim to propose an abdominal multi‐organ segmentation model on multi‐sequence MRI and CT. Consequently, the data heterogeneity is a major challenge. Therefore, the PFL is a promising avenue to solve this issue. Our PFL approach can be described as follows:

(2) $${W_{i}}^{l}=F\left({W_{i}}^{l-1}\right),l\in \left(1,E\right)$$

(3) $$W_{i}^{E}=\left[W_{Hi}^{E},W_{Ri}^{s}\right]$$

At each federated round *t*, the *i*‐th client trains *E* local epochs to obtain the best local personalized parameters. $W_{Hi}$ and $W_{Ri}$ are the current global representation communicated by the server. (l,s) are the local and global update epochs, respectively. F(·) is a generic notation for an update of the variable W.

In this way, the server updates the parameters of the global representation together. Meanwhile, the *i*‐th client learns its unique features locally. PFL can not only enhance the performance of local models but also decrease the calculation load of the global server. Nevertheless, another question is how to determine the global representation parameters and the personalized ones. In our study, U‐Net is exploited as the base network. As shown in Figure 1, the U‐Net can be divided into an encoder, a decoder, and a segmentation head. As proofed by FedRep,^21^ the segmentation head is used to map the representation to produce predicted values, which contain more personalized information. Therefore, in our work, the fully‐connected layers of segmentation map generation are regarded as personalized components. Additionally, inspired by SiloBN,^20^ BN is also used to separate local and domain‐invariant information.

(4) $$\mathrm{BN}\left(x\right)=\gamma \frac{x-\mu }{\sqrt{\sigma^{2}+\varepsilon }}+\beta$$

where ($\mu ,\sigma^{2}$) are calculated as the running means and variances of each channel computed across both spatial and batch dimensions, and (γ,β) are learned affine renormalization parameters. Consequently, we retain the local activation statistics ($\mu ,\sigma^{2}$) of BN and aggregate the trained renormalization (γ,β) at each federated round in this study.

Summarily, our local personalized parameters $W_{H}$ contain two parts: the parameters of fully‐connected layers for segmentation map generation (segmentation head in Figure 1) and the local activation statistics of BN.

### 2.3 Fourier transform attention aggregation in global server

After *E* rounds of local training, the global server collects the representation part $W_{R}$ from all the clients. However, except for the $W_{H}$ mentioned in section B, personalized information is also stored in the parameters of other layers. Therefore, by directly element‐wise averaging across $W_{R}$, the learned personalized information stored in it would be lost.

Inspired by the fact that low‐frequency components of parameters are the basis for the network capability,^25^ a novel weights aggregation mechanism based on Fast Fourier Transform and self‐Attention^26^ (FFTA) proposed to combine the low‐frequency components of $W_{R}$, while retaining the individual high‐frequency components.

For the *i*‐th client, the weight tensor $W_{Ri}\in R^{c_{\mathrm{in}}\times d_{1}\times d_{2}\times c_{\mathrm{out}}}$, where the spatial support of each kernel filter is $d_{1}\times d_{2}$. There are $c_{\mathrm{in}}$ input channels and $c_{\mathrm{out}}$ output channels. Then, $W_{Ri}$ can be reshaped into a 2D matrix 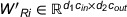 as for DCT‐domain multiplication, the detailed deduction process can be found elsewhere.^16^ $W_{Ri}^{\prime }=\left(W_{Ri}^{l},W_{Ri}^{h}\right)$, where $W_{Ri}^{l}$ and $W_{Ri}^{h}$ denote the low‐frequency component and high‐frequency component, as the following equations:

(5)

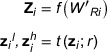

where f(·) denotes the Fourier Transform, t(·;r) denotes a threshold function that separates the low and high‐frequency components from z according to a hyper‐parameter. The center of 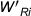 is set as the coordinate (0,0). For ease of calculation, the Fourier Transform and inverse Fourier Transform are computed by a fast algorithm, so‐called the FFT algorithm.^27^

**FIGURE 3:**
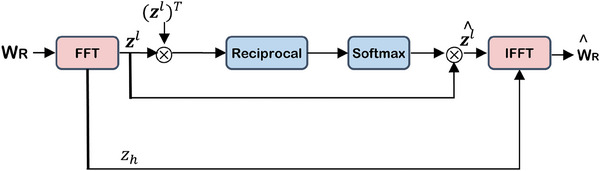
Self‐attention mechanism in global server aggregation.

The effectiveness of global server aggregation in the frequency domain has been proven by Chen et al.^28^ However, in their study, the heterogeneity of the data distribution has not been discussed. All clients had constant weight during the averaging in the global server. To tackle statistical heterogeneity and further improve the efficiency of network weight aggregation in the global server, the attention mechanism is harnessed to integrate the low‐frequency component of each client, which has been shown in Figure 3.

The attention mechanism can be regarded as an adaptive selection process based on the input feature.^29^ Inspired by the full attention network,^26^ the Self‐attention mechanism is utilized in this study, which can be described as follows:

(6) $$\hat{z^{l}}=softmax\left(\frac{1}{z^{l}{\left(z^{l}\right)}^{T}}\right)z^{l}$$

As shown in Figure 3, the global representation $W_{R}$ is divided into low‐frequency ($z^{l}$) and high‐frequency components ($z^{h}$) by Fourier Transform. Compared with the original Self‐attention algorithm, we take the reciprocal of $z^{l}{\left(z^{l}\right)}^{T}$ before the Softmax calculation. As mentioned in FedProx,^23^ different clients make different progress towards solving the local sub‐problems due to variable system conditions. The local updates would lead some sites towards the optima of their local objective as opposed to the global objective, which potentially hurts convergence or even causes the method to diverge. Therefore, the Self‐attention mechanism is used to assign higher weights for the model, which converges slowly, rather than computing the average of all the network weights directly. In this way, the issue of statistical heterogeneity is addressed by restricting the local updates without any need to manually set the weights for averaging.

After acquiring the $\hat{z^{l}}$ across all the clients, the aggregated frequency components for the *i*‐th client are calculated as:

(7) $$\hat{f}\left(W_{Ri}\right)=\left(z_{i}^{h},\frac{1}{K}\sum_{i=1}^{K}{\hat{z}}_{i}^{l}\right)$$

After applying the inverse Fourier Transform, the aggregated parameters $\hat{W_{Ri}}$ are obtained as:

(8) $$\hat{W_{Ri}}=f^{-1}\left(\hat{f}\left(W_{Ri}\right)\right)$$

Finally, the global representation weight $\hat{W_{Ri}}$ is sent to the *i*‐th client for the next round of training with weight $W_{i}=\left[W_{Hi}^{E},\hat{W_{Ri}}\right]$.

### 2.4 Dataset and implementations

1. MRI dataset: The Combined Healthy Abdominal Organ Segmentation (CHAOS) 2019 dataset comprises data from twenty patients for multi‐organ segmentation, including T1‐DUAL and T2‐SPIR abdominal MRI slices with ground‐truth delineations for the liver, right kidney, left kidney, and spleen. Notably, images of T1‐DUAL and T2‐SPIR sequences are not registered. This dataset contains 1270 image slices (647 T1‐DUAL, 623 T2‐SPIR), which were used for both abdominal multi‐organ segmentation experiments and evaluations. In pre‐processing, images were resampled into 1.62 × 1.62 mm (the median spacing), and then center cropped to 256 × 256 pixels.

**FIGURE 4:**
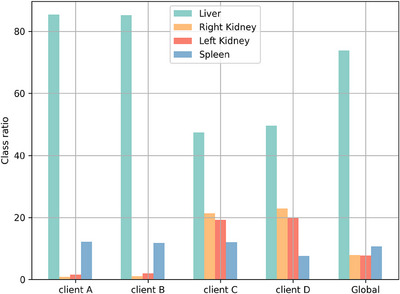
The class ratio of the T1‐DUAL dataset.

For each sequence of MRI, we distributed the dataset heterogeneously across four clients. The class ratio of the T1‐DUAL dataset in Figure 4 reveals the obvious imbalance between categories and clients. First, medical imaging is inherently unbalanced due to the size difference of abdominal organs. Second, there exist significant differences in the category distribution among clients.

2. CT dataset: We performed multi‐organ segmentation on abdominal images for the liver, duodenum, pancreas, and gall bladder. The dataset contains 4903 slices with 102 patients. All CT image samples were collected and authorized by Wuxi No.2 People's Hospital. These were labeled and confirmed by a senior radiologist from Wuxi No.2 People's Hospital. In pre‐processing, images were resampled into 1.0 × 1.0 mm (the median spacing), and then center cropped to 512 × 512 pixels.
3. Implementation Details: Our experiments were primarily conducted on NVIDIA GeForce RTX 3080 Ti GPUs in the Ubuntu operating system with the Pytorch framework. All models in the experiments were trained using the Adam optimizer with a learning rate of 10^−4^, and a batch size of 16. Before the model training, data augmentation methods such as random rotation and flipping were employed.

To speed up the training process, the convolutional neural network (CNN) backbone of all models was fine‐tuned from ImageNet pre‐trained weights. The epochs of local training and global training were 5 and 100, respectively. In other words, each local client was trained by 500 epochs. The hold‐out method and 5‐fold cross‐validation were adopted for model evaluation. The ratio between the amount of training, validation, and testing datasets was 8:1:1. Due to space limitations, we only presented the average evaluation metric values of each fold, detailed results of each fold; validation across different methods can be found in the Supplementary Material.

### 2.5 Loss function and evaluation metrics

In this paper, two commonly used metrics were exploited to evaluate the performance of segmentation: a region‐based metric, the Dice Similarity coefficient (DSC),^30^ and a boundary‐based metric, Average Symmetric Surface Distance (ASSD). The larger DSC and smaller ASSD represent the better segmentation results. The average scores of all local sites were used for assessment.

DSC is used to measure the similarity between the segmentation result and the ground truth, which is defined as:

(9) $$\mathrm{DSC}=\frac{2\mathrm{TP}}{2\mathrm{TP}+\mathrm{FP}+\mathrm{FN}}$$

ASSD is the average of all the distances from points on the boundary of the segmented region to the boundary of the ground truth, which is defined as:

(10) $$\begin{array}{cc} \mathrm{ASSD} & =\frac{1}{\left|S(O)|+|S(G)\right|}\left(\sum_{a\in S(O)}\min_{b\in S(G)}||a-b||\right.\\ & \;+\left.\sum_{b\in S(G)}\min_{a\in S(O)}||b-a||\right) \end{array}$$

We chose the combination of DSC and cross‐entropy^31^ as the loss function. The fusion segmentation loss is defined as:

(11) $$L_{seg}\left(G,O\right)=-\frac{1}{n}\sum_{i\in n}\sum_{j\in C}G_{i}^{j}\log\left(O_{i}^{j}\right)-\frac{2}{\left|C\right|}\sum_{j\in C}\frac{\sum_{i}O_{i}^{j}G_{i}^{j}}{\sum_{i}O_{i}^{j}+\sum_{i}G_{i}^{j}}$$

where TP, FP, and FN stand for true positive prediction, false positive prediction, and false negative prediction, respectively. *O* is the prediction, *G* is the ground truth with the one‐hot encoding, and 𝑖, 𝑗 denote the pixel and the class number of data, respectively. The boundary points on the predicted segmentation and the ground truth are denoted by (𝑂) and (𝐺).

## 3 RESULTS

### 3.1 Ablation studies

In this section, we systematically assess the efficacy of the proposed personalized federation learning algorithm, as illustrated in Table 1. The first row denotes our baseline, FedAvg. The intermediate models, each with one or two advanced modules, are designed as follows: (1) FedAvg with FFT: It incorporates the use of frequency domain aggregation at the global server. (2) FedAvg with Rep and BN (FPB in Table 1): This configuration amalgamates the personalized parameters from both the segmentation head layers and the local activation statistics of the BN. (3) PF‐Fed: the PFL with frequency domain aggregation at the global server. (4) PAF‐Fed (Ours): the PFL with frequency domain and Reverse Self‐attention aggregation at the global server.

**TABLE 1:** Ablation study on CHAOS 2019 MRI dataset.

	T1‐DUAL									
	DSC↑(%)					ASSD↓(mm)				
	Client A	Client B	Client C	Client D	Avg.	Client A	Client B	Client C	Client D	Avg.
FedAvg^18^	49.04	49.49	49.80	49.82	49.54	3.79	3.68	3.68	3.42	3.64
FedAvg + FFT	48.16	48.73	51.76	51.70	50.09	1.98	1.90	1.75	1.75	1.85
FPB	61.92	62.66	61.57	61.27	61.85	2.27	2.13	2.55	2.49	2.36
PF‐Fed	64.01	63.75	62.81	62.54	63.28	2.10	2.21	2.12	2.09	2.13
PAF‐Fed (Ours)	**64.04**	**64.13**	**63.72**	**63.74**	**63.91**	**1.54**	**1.50**	**1.71**	**1.69**	**1.61**

	T2‐SPIR									
	DSC↑(%)					ASSD↓(mm)				
	Client E	Client F	Client G	Client H	Avg.	Client E	Client F	Client G	Client H	Avg.
FedAvg^18^	62.49	63.39	63.32	61.80	62.75	2.56	2.51	2.50	2.70	2.57
FedAvg + FFT	63.56	62.16	66.32	66.04	64.52	1.75	1.82	2.34	2.25	2.04
FPB	63.84	66.31	65.28	65.50	65.23	2.90	2.63	2.63	2.69	2.71
PF‐Fed	78.97	79.82	79.70	79.94	79.61	**1.06**	1.09	1.28	1.28	1.18
PAF‐Fed (Ours)	**81.56**	**81.44**	**81.25**	**81.26**	**81.38**	1.12	**1.04**	**1.14**	**1.09**	**1.10**

*Notes*: Results are evaluated in DSC and ASSD scores. Bold text denotes the best results.

Abbreviations: ASSD, Average Symmetric Surface Distance; CHAOS, Combined Healthy Abdominal Organ Segmentation; DSC, Dice Similarity Coefficient; PAF‐FED, Personalized Federated Learning Framework.

Compared to the FedAvg model, the FFT manifested improvements, registering enhancements of 0.55% and 1.77% in the DSC scores for the T1‐DUAL and T2‐SPIR test datasets, respectively. As for PFL methods, the performance of FPB outstripped the FedAvg + FFT method. This superiority can be attributed to the feature learning garnered from the personalized layers. However, the ASSD was increased, which shows the worse boundary segmentation. Comparing PF‐Fed with our proposed PAF‐Fed framework, the introduction of Self‐attention into FFT gained improvements of 0.63% and 1.77% in the DSC scores for the T1‐DUAL and T2‐SPIR test datasets, respectively, demonstrating the advantage of Self‐attention modules.

Besides, centralized learning was also introduced for comparison, which can be seen as a performance upper limit. Specifically, the average DSC of centralized training in the T1‐DUAL and T2‐SPIR MRI datasets was 84.19% and 92.64%, respectively; the average ASSD of centralized training in the T1‐DUAL and T2‐SPIR MRI datasets was 0.62% and 0.38%. Due to the heterogeneity of datasets for each client, the centralized training performed much better.

Nonetheless, our proposed PAF‐Fed framework exhibited superior performance across both T1‐DUAL and T2‐SPIR datasets, demonstrating the advantage of both FFTA and FPB modules. The FPB module enhanced the performance by leveraging the learned knowledge from all clients, capitalizing on computational resources to execute multiple local updates, thereby refining local prediction capabilities. Additionally, the adaptive adjustment of the coefficients on frequency domain aggregation facilitated more robust and efficacious sharing of global knowledge.

### 3.2 Comparison experiments

Our proposed PAF‐Fed was compared with several state‐of‐the‐art PFL frameworks, encompassing FedBN,^19^ SiloBN,^20^ FedRep,^21^ LG‐FedAvg,^22^ and Fed‐Prox.^23^ A brief description of these methods is presented in the Introduction section. Besides, the conventional federation learning approach FedAvg,^18^ and local learning were also incorporated as reference methods. Local Train denotes that training of individual models leveraging their respective datasets. The results are shown in Table 2. All the models were trained and evaluated using the same environment, which has been presented in the Methods section, part D.

**TABLE 2:** Comparison study on CHAOS 2019 MRI dataset.

	T1‐DUAL									
	DSC↑(%)					ASSD↓(mm)				
Method	Client A	Client B	Client C	Client D	Avg.	Client A	Client B	Client C	Client D	Avg.
Local Train	32.17	34.72	35.70	35.66	34.56	8.70	4.50	3.17	2.35	4.68
FedAvg^18^	49.04	49.49	49.80	49.82	49.54	3.79	3.68	3.68	3.42	3.64
FedBN (2021)^19^	47.38	47.41	47.31	47.29	47.35	3.03	3.00	3.00	3.01	3.01
SiloBN (2020)^20^	50.45	51.25	52.40	52.41	51.63	1.92	1.83	1.87	1.89	1.88
FedRep (2021)^21^	61.77	60.18	59.71	59.69	60.34	2.17	2.16	2.24	2.25	2.21
LG‐FedAvg (2020)^22^	33.34	34.53	35.05	35.21	34.53	5.74	4.99	2.67	2.66	4.01
Fed‐Prox (2020)^23^	62.67	62.41	61.66	61.74	62.12	2.66	2.85	2.78	2.75	2.76
PAF‐Fed (Ours)	**64.04**	**64.13**	**63.72**	**63.74**	**63.91**	**1.54**	**1.50**	**1.71**	**1.69**	**1.61**

	T2‐SPIR									
	DSC↑(%)					ASSD↓(mm)				
Method	Client E	Client F	Client G	Client H	Avg.	Client E	Client F	Client G	Client H	Avg.
Local Train	32.79	32.53	49.15	45.43	39.97	6.29	7.30	4.39	6.28	6.07
FedAvg^18^	62.49	63.39	63.32	61.80	62.75	2.56	2.51	2.50	2.70	2.57
FedBN (2021)^19^	61.00	59.86	62.51	62.47	61.46	2.55	2.54	2.56	2.56	2.55
SiloBN (2020)^20^	65.84	65.75	65.71	65.72	65.76	2.25	2.32	3.20	3.08	2.71
FedRep (2021)^21^	80.98	80.74	80.01	79.94	80.42	1.24	1.29	1.54	1.54	1.40
LG‐FedAvg (2020)^22^	35.40	34.72	48.42	43.16	40.43	5.65	6.00	4.87	6.35	5.72
Fed‐Prox (2020)^23^	79.98	79.39	78.36	78.39	79.03	1.69	1.39	1.24	1.31	1.41
PAF‐Fed (Ours)	**81.56**	**81.44**	**81.25**	**81.26**	**81.38**	**1.12**	**1.04**	**1.14**	**1.09**	**1.10**

*Notes*: Results are evaluated in DSC and ASSD scores. Bold text denotes the best results.

Abbreviations: ASSD, Average Symmetric Surface Distance; CHAOS, Combined Healthy Abdominal Organ Segmentation; DSC, Dice Similarity Coefficient; PAF‐FED, Personalized Federated Learning Framework.

#### 3.2.1 MRI dataset

For the T1‐DUAL dataset, FedAvg recorded an average DSC of 49.54% and an average ASSD of 3.64, setting a baseline within the FL methods. Comparative analysis revealed that FedAvg enhanced segmentation outcomes by 14.98% on average of DSC and 1.04 on average of ASSD relative to Local Train. This enhancement underscores the potent synergy achieved through FL, empowering clients with limited datasets to acquire robust deep model architectures. FedBN even had worse performance than FedAvg, potentially due to the training collapse. Notably, SiloBN outperformed the FedBN with a remarkable increase of 4.28% in average DSC. This improvement validated that regarding local activation statistics of BN as personalized parameters could efficiently address the retrogress in FedBN. Furthermore, FedRep could improve the DSC more effectively by exploring shared representations and personalizing the prediction layers.

In contrast, our PAF‐Fed framework achieved overwhelming performance across all clients, with the best average DSC of 63.91% and an average ASSD of 1.61. Relative to FedRep, which integrated local representation learning with global model learning, the PAF‐Fed framework achieved improved performance by 3.57% in average DSC and 0.60 in average ASSD, demonstrating the salient efficacy of our FFTA algorithm. In conclusion, these comparisons confirmed the advantages of the PAF‐Fed framework over state‐of‐the‐art works within the T1‐DUAL MRI dataset.

Results on the T2‐SPIR dataset further support the superior performance of the PAF‐Fed framework. Notably, segmentation complexities within T2‐SPIR were lower than T1‐DUAL, since the Local Train had reached an average DSC of 39.97% in T2‐SPIR, which was 5% higher than T1‐DUAL. All the FL algorithms performed better than local training for joint learning through multiple clients. However, PFL methods such as FedBN and LG‐FedAVG obtained worse DSC than FedAVG. The possible reason might be the wrong personalized layer selection. LG‐FedAVG retained too much information on local clients; thus, the model performance was close to that of local training. In comparison, PAF‐Fed consistently outperformed across these state‐of‐the‐art methods in both average scores of DSC and ASSD, which demonstrated the advancement of our proposed PAF‐Fed.

#### 3.2.2 CT dataset

To demonstrate the generalizability to other datasets, we further compared the proposed method with SOTA methods for abdominal multi‐organ segmentation on private CT datasets. The compared methods were similar to those in the comparison study on the CHAOS 2019 dataset.

Table 3 reports the comparison results. Due to more training data, the difficulty of model training was reduced, and the Local Train had already reached an average DSC of 73.57%. Therefore, FedAVG showed a close performance compared with Local Train, for data of each client was enough to train a satisfactory model. Compared with the baseline FedAVG, SiloBN obtained an 8.73% increase in average DSC, by alleviating the data heterogeneity among clients with specific normalization statistics. Our method achieved the outperforming segmentation results on most clients. It demonstrated the superiority of our proposed PAF‐Fed, even in the situation that each local client could provide enough data to train their segmentation model.

**TABLE 3:** Comparison study on private CT dataset.

	DSC↑(%)					ASSD↓(mm)				
Method	Client I	Client J	Client K	Client L	Avg.	Client I	Client J	Client K	Client L	Avg.
Local Train	72.20	73.85	73.80	74.44	73.57	31.46	15.76	12.23	32.74	23.05
FedAvg^18^	75.75	71.92	75.17	76.23	74.77	21.51	18.50	13.33	22.31	18.91
FedBN (2021)^19^	79.24	81.07	70.57	79.55	77.61	19.08	13.47	16.03	22.39	17.74
SiloBN (2020)^20^	84.65	83.59	84.80	80.98	83.50	23.66	14.60	20.56	23.81	20.66
FedRep (2021)^21^	**85.49**	84.74	**86.13**	80.21	84.14	**14.68**	12.85	11.80	21.55	15.22
LG‐FedAvg (2020)^22^	67.07	75.29	74.17	78.25	73.70	47.79	25.10	13.42	19.33	26.41
Fed‐Prox (2020)^23^	80.40	82.00	81.34	80.40	81.03	23.14	24.87	22.04	18.06	22.03
PAF‐Fed (Ours)	85.15	**85.42**	85.87	**85.56**	**85.50**	17.51	**8.53**	**11.76**	**17.51**	**13.83**

*Notes*: Results are evaluated in DSC and ASSD scores. Bold text denotes the best results.

Abbreviations: ASSD, Average Symmetric Surface Distance; DSC, Dice Similarity Coefficient; PAF‐FED, Personalized Federated Learning Framework.

### 3.3 Visual comparison

**FIGURE 5:**
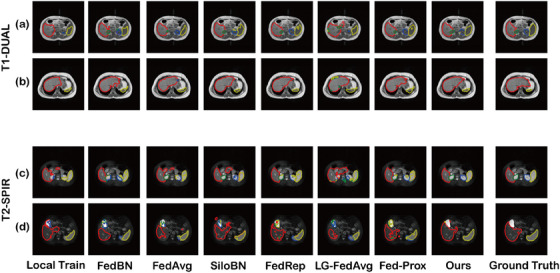
Visualization of the abdominal multi‐organ segmentation results for the CHAOS 2019 MRI dataset. Red—Liver, Green—Right Kidney, Blue—Left Kidney, Yellow—Spleen. CHAOS, Combined Healthy Abdominal Organ Segmentation.

**FIGURE 6:**
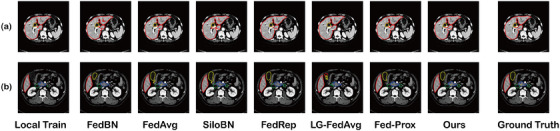
Visualization of the abdominal multi‐organ segmentation results for private CT dataset. Red—Liver, Green—Duodenum, Blue—Pancreas, Yellow—Gall Bladder.

Figures 5 and 6 provide visual representations of abdominal multi‐organ segmentation results by our proposed method and other FL methods.

Figure 5 provides a visual representation of four distinct samples. Figure 5a,b are from the T1‐DUAL dataset, and Figure 5c,d are from the T2‐SPIR dataset, respectively. To ensure an unbiased and direct comparison, the segmentation outcomes did not undergo any post‐processing.

As shown in Figure 5a, almost all the state‐of‐the‐art methods exhibited suboptimal performance, notably evident in the poor segmentation for the right kidney and left kidney. As for Figure 5b, the main difficulty lies in the segmentation of the apex of the liver. No approach successfully segmented the whole liver area. In general, within the T1‐DUAL dataset, the PFL architectures could improve the robustness of segmentation. However, the target was easy to determine in Figure 5c, which has been proven by quantitative segmentation results of these four organs (Table 4). Nonetheless, certain methods led to over‐segmentation, as shown in the results of FedAVG and SiloBN. As for Figure 5d, the white artifacts in the upper‐left corner resulted in huge difficulty in recognition. Several methods incorrectly segmented this part into organs. In contrast, our proposed PAF‐Fed consistently yielded optimal segmentation results, underscoring its robustness and efficacy across the T1‐DUAL and T2‐SPIR datasets.

**TABLE 4:** Comparison study to each organ.

	T1‐DUAL				T2‐SPIR				CT			
Method	Liver	RK	LK	Spleen	Liver	RK	LK	Spleen	Liver	Dou	Pan	GB
Local Train	54.78	13.38	25.87	44.23	51.43	25.46	31.50	51.50	94.55	57.94	69.15	72.66
FedAvg^18^	63.30	37.01	44.87	52.96	63.68	60.28	58.97	68.08	95.21	60.75	73.04	70.06
FedBN(2021)^19^	66.60	37.55	39.54	45.70	65.20	52.39	57.30	70.96	95.01	69.53	72.09	73.80
SiloBN(2020)^20^	66.81	44.56	43.80	51.35	66.45	62.10	61.55	72.93	95.47	76.93	82.53	79.08
FedRep(2021)^21^	**74.19**	48.81	50.15	**68.21**	80.59	77.71	77.02	86.36	96.16	77.89	82.84	**79.67**
LG‐FedAVG(2021)^22^	51.54	17.71	24.97	43.91	53.42	25.38	30.37	52.54	94.95	58.56	71.23	70.05
Fed‐Prox(2020)^23^	62.67	62.41	61.66	61.74	80.02	78.71	74.32	83.06	96.31	71.81	77.96	78.05
PAF‐Fed(Ours)	64.04	**64.13**	**63.72**	63.74	**81.50**	**79.84**	**77.28**	**86.89**	**97.26**	**83.32**	**86.49**	74.93

*Notes*: Results are evaluated in the DSC score. Bold text denotes the best results.

Abbreviations: Dou, Duodenum; DSC, Dice Similarity Coefficient; GB, Gall bladder; LK, Left kidney; PAF‐FED, Personalized Federated Learning Framework; Pan, Pancreas; RK, Right kidney.

In terms of our private CT dataset, the multi‐organ segmentation in CT scans is much easier than in MRI, due to the high‐density resolution of CT. Figure 6 demonstrates that the main difficulties of multi‐organ segmentation are in the small organ and organ boundary, such as the segmentation of the gall bladder in Figure 6b. Table 4 shows the effectiveness of our proposed methods in improving segmentation performance, especially for small organs like kidneys, duodenum, and pancreas. In terms of the performance of the liver and spleen in MRI, the average DSC of our method was slightly lower than that of FedRep. The reason could be that the FFTA module assigned higher weights for the model, which converged slowly, which led to the results of each organ being more balanced.

### 3.4 Analysis of hyper‐parameters

**FIGURE 7:**
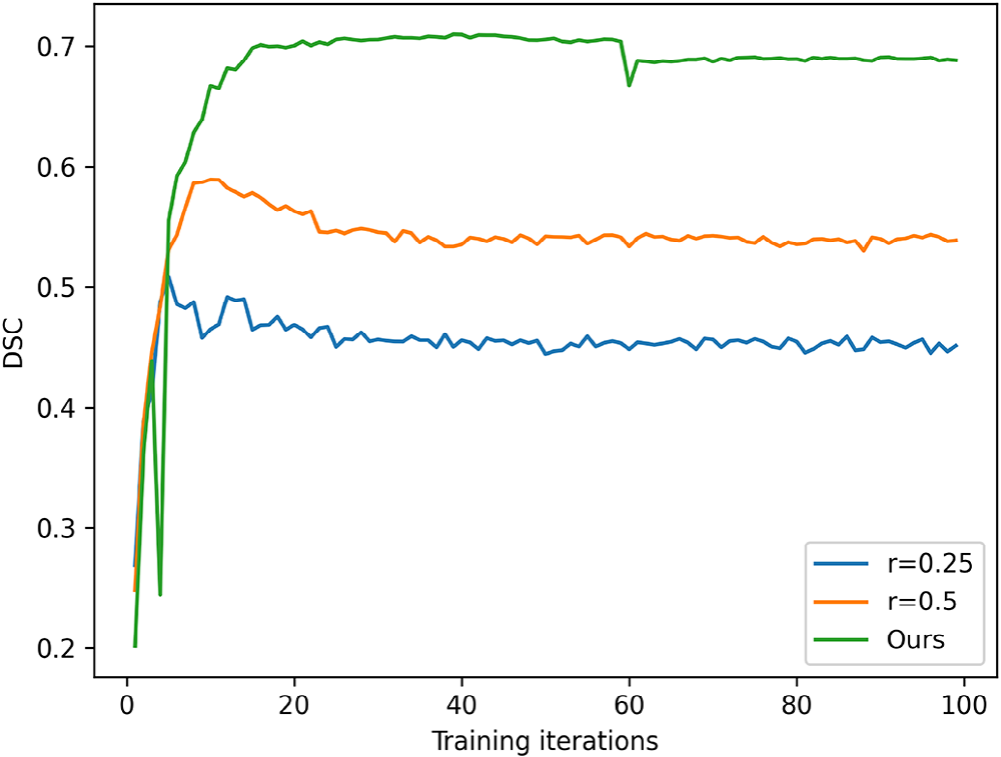
Comparison of learning curves at different values of r in Equation (5).

To determine the optimal *r*‐value in Equation (5), we chose *r* = 0.25, 0.5, and the adaptive adjustment method proposed by Chen et al.^28^ for model training, respectively. As for the adaptive adjustment method, $r=r_{0}+\frac{r_{1}-r_{0}}{T}t$, where *r_0_* = 0 and *r_1_* = 0.5 were the initial and terminated low‐frequency thresholds. The change of DSC in FL model training based on the T1‐DUAL dataset is demonstrated in Figure 7. Therefore, we used $r=r_{0}+\frac{r_{1}-r_{0}}{T}t$ in this work.

## 4 DISCUSSION

Abdominal segmentation is crucial for various clinical applications, including automated diagnosis, surgical planning and navigation, monitoring disease progression, and facilitating personalized treatment strategies. It creates preliminary segmentation automatically, which can guide the references and evaluations for radiologists. Despite advancements in DL‐based segmentation methods, there remains a significant dependency on extensive labeled datasets for effective training. Following the standard FL frameworks, our proposed method can benefit from the large effective training set without sharing the raw data between clients. The PFL on BN and segmentation head enhances the performance by leveraging the learned knowledge from all clients, meanwhile using the computational power of clients to run multiple local updates for learning their local prediction heads. Concurrently, Fourier Transform Attention aggregation in the global server could achieve more stable and effective global knowledge gathering. Moreover, PAF‐Fed was evaluated on the CHAOS 2019 MRI and private CT datasets, manifesting superior average DSC and ASSD relative to other FL architectures.

Despite achieving generally superior performance in abdominal multi‐organ segmentation, distinguishing accuracy on small organs such as kidneys still needs improvement. A DSC of 80% or higher is often considered accepted to ensure reliable identification of abdominal organs.^8^ However, none of the automatic segmentation results with kidneys can meet this threshold. A possible explanation for this limitation lies in the class imbalance of multi‐organ segmentation. In imbalanced datasets, the dominant categories with more samples would overwhelm the rare categories, which severely degrade the performance of biased networks. Therefore, as a prospective avenue of investigation, we plan to further improve the segmentation accuracy of small organs by dealing with the challenge of class imbalance.

## 5 CONCLUSION

In this study, we introduced an innovative PFL framework called PAF‐Fed for abdominal multi‐organ segmentation. On the client side, a new PFL on BN and segmentation heads was utilized to combine local representation learning with FL of global models. On the server side, the Fourier Transform Attention aggregation was employed to integrate the global knowledge from low‐frequency to high‐frequency gradually. The proposed method was evaluated on the MRI and CT datasets, achieving an average DSC of 72.65% and 85.50%, respectively. Plenty of experiments substantiate the efficacy and remarkable advantages of our proposed PAF‐Fed framework over state‐of‐the‐art works.

## AUTHOR CONTRIBUTIONS

Junzhong Zou conceived and designed the study. Hao Fu and Jian Zhang developed the experiments and wrote the manuscript. Lanlan Chen engaged in revisions.

## CONFLICT OF INTEREST STATEMENT

The authors declare that there is no conflict of interest that could be perceived as prejudicing the impartiality of the research reported.

## Supporting information



Supporting Information
