# Supplementary material for: Personalized federated learning for abdominal multi‐organ segmentation based on frequency domain aggregation
Source: J Appl Clin Med Phys. 2024 Dec 5;26(2):e14602. doi: 10.1002/acm2.14602 (PMC11799920; doi:10.1002/acm2.14602)
Supplement: Supplementary file 1 — Supporting Information [file ACM2-26-e14602-s001.pdf]

Supplementary material

Results of 5-fold cross-validation experiments.

1. Local train

T1-DUAL DSC

		Liver	Right Kidney	Left Kidney	Spleen	Mean
FOLD1	client A	49.97	0	29.96	42.85	30.7
	client B	58.77	0.02	31.6	54.05	36.11
	client C	57.63	24.99	21.72	41.11	36.36
	client D	58.86	24.91	22.94	45.05	37.94
FOLD2	client A	58.7	4.16	20.97	50.81	33.66
	client B	65.44	2.18	35.09	56.67	39.84
	client C	55.3	24.3	24.16	37.25	35.25
	client D	53.1	26.09	24.34	34.32	34.46
FOLD3	client A	45.01	0	28.07	37.95	27.76
	client B	46.69	0.12	30.08	40.88	29.44
	client C	53.55	28.69	26.22	37.44	36.47
	client D	53.05	24.06	28.49	32.01	34.4
FOLD4	client A	58.12	0	32.14	55.78	36.51
	client B	60.22	0	26.15	53.96	35.08
	client C	53.89	22.91	22.21	36.97	33.99
	client D	56.6	25.3	24.69	38.16	36.19
FOLD5	client A	50.2	6.28	11.23	61.19	32.22
	client B	50.24	3.35	30.24	48.74	33.14
	client C	55.41	25.26	23.07	41.93	36.42
	client D	54.8	25.04	24.01	37.41	35.32

T1-DUAL ASSD

		Liver	Right Kidney	Left Kidney	Spleen	Mean
FOLD1	client A	1.64	69.07	3.88	6.68	20.32
	client B	1.23	12.83	2.14	3.94	5.03
	client C	1.27	2.87	2.42	1.61	2.04
	client D	1.3	2.75	2.21	1.86	2.03
FOLD2	client A	1.36	5.51	12.75	4.63	6.06
	client B	1.63	5.9	8.63	1.6	4.44
	client C	1.71	4.03	10.06	2.91	4.68
	client D	1.76	2.45	3.55	2.91	2.67
FOLD3	client A	4.9	10.09	2.43	3.85	5.32
	client B	6.7	7.94	2.45	7.01	6.03
	client C	1.55	2.93	3.29	3.51	2.82
	client D	1.35	3.18	2.22	2.16	2.23

FOLD4	client A	1.4	0	2.75	0.86	1.25
	client B	1.52	0	3.2	1.19	1.48
	client C	1.6	2.95	6.63	1.96	3.29
	client D	1.71	2.95	2.34	2.15	2.29
FOLD5	client A	3.06	9.38	28.82	0.99	10.56
	client B	1.48	4.13	14.72	1.76	5.52
	client C	1.55	3.07	5.58	1.78	3
	client D	1.67	2.34	3.27	2.93	2.55

T2-SPIR DSC

		Liver	Right Kidney	Left Kidney	Spleen	Mean
FOLD1	client A	56.67	0.03	10.6	59.74	31.76
	client B	53.41	0	0	68.52	30.48
	client C	48.01	50.64	52.26	48.59	49.87
	client D	46.92	53.22	52.09	43.08	48.83
FOLD2	client A	58.49	0	6.13	63.85	32.12
	client B	55.64	0	8.8	66.7	32.78
	client C	47.69	48.72	57.41	50.29	51.03
	client D	45.81	49.06	52.14	39.81	46.71
FOLD3	client A	55.42	7.67	13.87	70.28	36.81
	client B	53.47	1.54	8.87	69.95	33.46
	client C	46.14	41.62	49.73	49.78	46.82
	client D	44.12	50.93	48.65	39.69	45.85
FOLD4	client A	59.32	0.42	8.56	39.23	26.89
	client B	57.27	0.15	19.72	41.19	29.59
	client C	55.62	51.57	48.28	44.36	49.96
	client D	48.16	52.6	52.65	15.83	42.31
FOLD5	client A	57.58	0	15.62	72.27	36.37
	client B	52.66	0	28.38	64.36	36.35
	client C	44.01	49.68	47.89	50.63	48.05
	client D	42.15	51.34	48.39	31.88	43.44

T2-SPIR ASSD

		Liver	Right Kidney	Left Kidney	Spleen	Mean
FOLD1	client A	1.21	11.39	3.12	7.2	5.73
	client B	1.62	16.53	28.72	1.13	12
	client C	1.67	4.27	2.98	2.61	2.88
	client D	1.98	4.27	3.65	3.78	3.42
FOLD2	client A	1.48	10.32	6.63	4	5.6
	client B	1.26	0	5.51	1.84	2.15
	client C	2.48	6.09	4.23	7.21	5

	client D	2.7	7.18	7.02	6.58	5.87
FOLD3	client A	1.26	5.75	4.67	1.73	3.35
	client B	1.57	8.93	5.49	4.41	5.1
	client C	2.24	8.04	2.09	1.97	3.59
	client D	2.1	3.93	4.54	3.65	3.56
FOLD4	client A	4.91	6.16	20.13	19.58	12.7
	client B	3.6	8.88	9.48	28.46	12.61
	client C	1.59	7.99	6.17	8.12	5.97
	client D	2.42	7.64	5.59	40.75	14.1
FOLD5	client A	1.76	10.27	3.04	1.23	4.07
	client B	1.77	12.29	3.11	1.43	4.65
	client C	2.35	7.06	4.15	4.5	4.52
	client D	3.24	6.03	3.73	4.8	4.45

2. FedAVG

T1-DUAL DSC

		Liver	Right Kidney	Left Kidney	Spleen	Mean
FOLD1	client A	71.85	46.55	47.69	49.72	53.95
	client B	70.48	45.57	48.02	49.47	53.38
	client C	73.21	48.14	48.61	50.59	55.14
	client D	73.21	48.14	48.61	50.59	55.14
FOLD2	client A	69.23	22.33	43.42	59.3	48.57
	client B	69.23	22.33	43.42	59.3	48.57
	client C	69.2	22.24	43.41	59.27	48.53
	client D	69.23	22.33	43.42	59.3	48.57
FOLD3	client A	57.71	41.97	54.14	51.01	51.21
	client B	57.73	42	54.38	51.13	51.31
	client C	57.73	41.93	53.92	50.9	51.12
	client D	57.74	41.93	53.99	51.02	51.17
FOLD4	client A	58.07	44.67	38.63	43.45	46.2
	client B	59.36	42.86	42.22	45.7	47.54
	client C	59.36	42.86	42.22	45.7	47.54
	client D	59.36	42.86	42.22	45.7	47.54
FOLD5	client A	56.74	29.26	36.06	58.95	45.25
	client B	58.85	30.71	37.69	59.36	46.65
	client C	58.85	30.76	37.71	59.38	46.67
	client D	58.85	30.76	37.71	59.38	46.67

T1-DUAL ASSD

		Liver	Right Kidney	Left Kidney	Spleen	Mean
FOLD1	client A	0.65	1.11	1.26	1.28	1.08
	client B	0.67	1.13	1.26	1.32	1.09
	client C	0.66	1.08	1.23	1.26	1.06
	client D	0.66	1.08	1.23	1.26	1.06
FOLD2	client A	1.01	1.88	1.44	0.88	1.3
	client B	1.01	1.88	1.44	0.88	1.3
	client C	1.01	1.89	1.44	0.88	1.31
	client D	1.01	1.88	1.44	0.88	1.3
FOLD3	client A	2.97	1.55	1.66	2.03	2.05
	client B	2.6	1.53	1.82	1.84	1.95
	client C	3.01	1.55	1.67	2.04	2.07
	client D	3.01	1.56	1.67	2.04	2.07
FOLD4	client A	1	1.39	1.43	1.43	1.31
	client B	1.04	1.47	1.31	1.95	1.44
	client C	1.04	1.47	1.31	1.95	1.44
	client D	1.04	1.47	1.31	1.95	1.44
FOLD5	client A	1.13	4.17	1.45	1.35	2.03
	client B	1.12	3.7	1.4	1.4	1.9
	client C	1.12	3.69	1.4	1.4	1.9
	client D	1.12	3.69	1.4	1.4	1.9

T2-SPIR DSC

		Liver	Right Kidney	Left Kidney	Spleen	Mean
FOLD1	client A	58.13	40.11	43.1	61.86	50.8
	client B	58.12	40.09	43.1	61.86	50.79
	client C	58.11	40.09	43.04	61.83	50.77
	client D	58.13	40.11	43.11	61.86	50.8
FOLD2	client A	63.86	70.89	70.43	73.31	69.62
	client B	65.41	73.06	73.19	74.63	71.57
	client C	65.41	73.06	73.19	74.63	71.57
	client D	64.05	71.36	71.09	73.7	70.05
FOLD3	client A	62.57	63.45	64.61	72.58	65.8
	client B	67.37	66.35	64.44	71.32	67.37
	client C	67.37	66.36	64.44	71.32	67.37
	client D	67.37	66.35	64.44	71.32	67.37
FOLD4	client A	69.57	70.2	54.49	61.9	64.04
	client B	70.36	71.25	54.6	62.62	64.71
	client C	71.76	72.37	56.94	63.37	66.11
	client D	64.74	63.44	51.74	60.09	60
FOLD5	client A	60.94	54.44	61.9	71.42	62.17

	client B	61.41	54.5	62.53	71.61	62.51
	client C	59.42	54.01	59.52	70.2	60.79
	client D	59.42	54.01	59.52	70.2	60.79

T2-SPIR ASSD

		Liver	Right Kidney	Left Kidney	Spleen	Mean
FOLD1	client A	0.99	9.55	9.81	3.67	6.01
	client B	0.99	9.53	9.8	3.67	6
	client C	0.99	9.5	9.76	3.66	5.98
	client D	0.99	9.55	9.81	3.67	6
FOLD2	client A	0.88	2.05	1.26	1.43	1.41
	client B	0.96	1.97	1.14	1.28	1.34
	client C	0.96	1.97	1.14	1.28	1.34
	client D	0.87	2.03	1.31	1.41	1.41
FOLD3	client A	1.04	3.88	2.81	1.95	2.42
	client B	0.99	3.31	2.98	2.11	2.35
	client C	0.99	3.31	2.98	2.11	2.35
	client D	0.99	3.31	2.98	2.11	2.35
FOLD4	client A	1.19	0.99	1.64	1.82	1.41
	client B	1.21	0.92	1.47	1.59	1.3
	client C	1.23	1.41	1.47	1.32	1.36
	client D	1.53	1.34	2.89	3.46	2.31
FOLD5	client A	1.04	2.2	1.13	1.81	1.54
	client B	1.05	2.24	1.1	1.86	1.56
	client C	0.97	2.2	1.19	1.45	1.45
	client D	0.97	2.2	1.19	1.45	1.45

3. FedBN

T1-DUAL DSC

		Liver	Right Kidney	Left Kidney	Spleen	Mean
FOLD1	client A	64.2	39.95	42.94	57.08	51.04
	client B	64.27	39.93	42.86	56.93	51
	client C	64.25	39.38	42.76	56.95	50.83
	client D	64.19	38.92	42.86	56.92	50.72
FOLD2	client A	69.86	36.33	45.59	45.22	49.25
	client B	69.99	36.19	45.57	45.49	49.31
	client C	70.03	36.08	45.3	44.69	49.02
	client D	70.14	35.9	45.56	44.9	49.13
FOLD3	client A	69.54	42	49.9	49.5	52.73

	client B	69.57	41.71	49.99	49.46	52.68
	client C	69.98	41.55	49.79	49.61	52.73
	client D	69.97	41.47	49.66	49.44	52.63
FOLD4	client A	65.27	40.56	31.75	34.18	42.94
	client B	65.42	40.68	31.69	34.39	43.05
	client C	65.5	40.67	31.46	34.29	42.98
	client D	65.66	40.58	31.41	34.24	42.97
FOLD5	client A	63.35	29.49	28.02	42.83	40.92
	client B	63.37	29.78	28.13	42.68	40.99
	client C	63.71	29.94	27.78	42.58	41
	client D	63.68	29.97	27.71	42.67	41.01

T1-DUAL ASSD

		Liver	Right Kidney	Left Kidney	Spleen	Mean
FOLD1	client A	0.91	1.56	1.83	1	1.33
	client B	0.91	1.56	1.71	1	1.3
	client C	0.89	1.6	1.83	0.99	1.33
	client D	0.88	1.62	1.92	1	1.36
FOLD2	client A	0.82	2.03	1.16	3.86	1.97
	client B	0.82	2.04	1.17	3.76	1.95
	client C	0.81	2.13	1.17	3.81	1.98
	client D	0.81	2.08	1.15	3.73	1.94
FOLD3	client A	0.79	1.85	1.06	1.86	1.39
	client B	0.79	1.91	1.09	1.84	1.41
	client C	0.78	1.86	1.09	1.88	1.4
	client D	0.78	1.86	1.04	1.89	1.39
FOLD4	client A	0.85	2.44	1.49	18.97	5.94
	client B	0.84	2.4	1.49	18.68	5.85
	client C	0.83	2.42	1.5	18.69	5.86
	client D	0.82	2.38	1.5	18.96	5.91
FOLD5	client A	1.2	3.08	6.91	6.79	4.5
	client B	1.22	3.05	6.89	6.76	4.48
	client C	1.21	3.03	6.92	6.63	4.45
	client D	1.21	3.07	6.93	6.62	4.46

T2-SPIR DSC

		Liver	Right Kidney	Left Kidney	Spleen	Mean
FOLD1	client A	60.63	55.73	56.82	73.29	61.62
	client B	60.6	55.01	57.28	73.35	61.56
	client C	57.95	45.68	49.76	72.65	56.51
	client D	58.12	46.62	53.24	72.44	57.6

FOLD2	client A	64.65	23.99	63.03	73.01	56.17
	client B	64.45	19.08	63.08	72.8	54.85
	client C	65.05	40.74	60.08	73.29	59.79
	client D	64.93	40.44	59.22	73.36	59.49
FOLD3	client A	69.6	66.17	56.84	70.87	65.87
	client B	69.38	64.14	55.24	70.02	64.69
	client C	70.29	58.54	56.26	69.55	63.66
	client D	70.31	58.8	55.04	68.65	63.2
FOLD4	client A	66.31	56.1	65.48	72.48	65.09
	client B	66.22	50.34	65.56	72.05	63.54
	client C	68.81	69.87	66.66	73.38	69.68
	client D	68.66	69.66	66.89	73.33	69.64
FOLD5	client A	62.85	48.78	44.71	68.72	56.26
	client B	62.36	42.66	44.65	69	54.67
	client C	66.39	67.78	53.46	63.98	62.9
	client D	66.35	67.65	52.69	63.05	62.44

T2-SPIR ASSD

		Liver	Right Kidney	Left Kidney	Spleen	Mean
FOLD1	client A	0.84	1.49	2.33	4.63	2.32
	client B	0.84	1.5	2.36	4.5	2.3
	client C	0.82	1.59	2.28	4.71	2.35
	client D	0.83	1.58	2.29	4.67	2.34
FOLD2	client A	1.85	1.7	4.41	1.48	2.36
	client B	1.81	1.65	4.14	1.5	2.27
	client C	1.85	1.71	4.32	1.52	2.35
	client D	1.79	1.7	4.31	1.51	2.33
FOLD3	client A	1.07	10.24	1.82	2.31	3.86
	client B	1.06	10.41	1.82	2.32	3.9
	client C	1.05	10.2	1.88	2.36	3.87
	client D	1.06	10.21	1.89	2.36	3.88
FOLD4	client A	1.36	2.38	3.48	1.3	2.13
	client B	1.34	2.38	3.51	1.32	2.14
	client C	1.33	2.35	3.62	1.35	2.16
	client D	1.31	2.33	3.58	1.33	2.14
FOLD5	client A	0.79	4	2.44	1.05	2.07
	client B	0.79	4.04	2.44	1.1	2.09
	client C	0.78	4.09	2.48	0.98	2.08
	client D	0.78	4.05	2.54	1	2.1

4. SiloBN

T1-DUAL DSC

		Liver	Right Kidney	Left Kidney	Spleen	Mean
FOLD1	client A	67.76	56.78	47.22	55.26	56.75
	client B	69.09	58.91	49.64	57.39	58.76
	client C	71.18	58.14	50.18	56.79	59.07
	client D	71.18	58.14	50.18	56.79	59.07
FOLD2	client A	63.35	30.46	33.25	46.16	43.31
	client B	63.95	30.67	33.93	46.22	43.69
	client C	67.39	33.66	34.7	49.72	46.37
	client D	67.55	33.78	34.79	49.7	46.46
FOLD3	client A	63.3	46.4	51.16	51.6	53.11
	client B	61.43	42.91	50.5	50.57	51.35
	client C	64.69	46.3	52.01	53.27	54.07
	client D	64.69	46.3	52.01	53.27	54.06
FOLD4	client A	61.94	34.45	44.74	44.94	46.52
	client B	64.37	42.14	45.49	47.42	49.85
	client C	64.35	42.26	45.47	47.44	49.88
	client D	64.37	42.14	45.49	47.41	49.85
FOLD5	client A	71.43	46.91	38.67	53.25	52.57
	client B	71.4	46.95	38.84	53.27	52.62
	client C	71.4	46.95	38.84	53.27	52.62
	client D	71.4	46.95	38.84	53.27	52.62

T1-DUAL ASSD

		Liver	Right Kidney	Left Kidney	Spleen	Mean
FOLD1	client A	1.82	2.13	2.14	1.52	1.9
	client B	1.36	2.62	1.7	1.61	1.83
	client C	1.07	1.53	1.96	1.86	1.61
	client D	1.21	1.38	1.83	1.56	1.5
FOLD2	client A	1.49	1.93	1.75	1.47	1.66
	client B	1.1	1.53	1.61	1.46	1.42
	client C	0.92	1.9	4.05	1.72	2.15
	client D	0.91	2.06	3	1.25	1.8
FOLD3	client A	1.36	1.71	1.78	2.79	1.91
	client B	1.51	1.8	1.69	1.76	1.69
	client C	0.85	1.49	2.05	2.2	1.65
	client D	1.16	2.22	2.42	1.71	1.88
FOLD4	client A	1.03	1.33	3.59	1.14	1.77
	client B	0.93	1.38	3.27	1.16	1.68
	client C	1.04	2.21	1.72	1.82	1.7
	client D	1.12	2.31	1.77	2.32	1.88
FOLD5	client A	1.05	4.33	3.16	0.91	2.36

	client B	0.96	4.67	3.22	1.25	2.53
	client C	0.84	2.56	3.58	1.95	2.23
	client D	0.78	3.05	3.72	1.99	2.39

T2-SPIR　DSC

		Liver	Right Kidney	Left Kidney	Spleen	Mean
FOLD1	client A	66.16	70.06	63.65	70.6	67.62
	client B	66.15	69.97	63.68	70.76	67.64
	client C	66.17	69.52	63.18	70.59	67.37
	client D	66.24	69.38	63.29	70.48	67.35
FOLD2	client A	61.32	74.8	62.16	76.47	68.69
	client B	61.3	74.87	62.16	76.68	68.75
	client C	61.22	74.68	61.71	76.56	68.54
	client D	61.26	74.66	61.46	76.72	68.52
FOLD3	client A	68.42	45.52	58.75	63.67	59.09
	client B	68.25	45.32	58.48	63.21	58.81
	client C	68.42	45.93	58.54	63.47	59.09
	client D	68.38	45.94	58.87	63.76	59.24
FOLD4	client A	65.75	54.97	57.24	74.14	63.02
	client B	65.83	54.79	57.03	73.9	62.89
	client C	65.73	54.53	57.08	74.22	62.89
	client D	65.68	54.8	56.85	74.03	62.84
FOLD5	client A	70.76	66.04	66.57	79.72	70.77
	client B	70.6	65.99	66.61	79.48	70.67
	client C	70.72	64.99	66.86	80.16	70.68
	client D	70.64	65.14	66.83	80.02	70.66

T2-SPIR　ASSD

		Liver	Right Kidney	Left Kidney	Spleen	Mean
FOLD1	client A	1.04	4	6.17	1.49	3.18
	client B	1.1	3.89	5.75	1.53	3.07
	client C	0.85	7.24	11.62	1.38	5.27
	client D	0.82	6.72	9.29	1.26	4.52
FOLD2	client A	0.87	6.97	1.47	2.84	3.03
	client B	0.89	7.04	1.5	3.15	3.14
	client C	0.72	7.51	3.36	3.48	3.77
	client D	0.71	7.49	3.7	3.22	3.78
FOLD3	client A	0.65	2.5	2.23	3.36	2.18
	client B	0.66	2.42	2.11	3.85	2.26
	client C	0.57	5.88	3.85	2.22	3.13
	client D	0.57	6.3	3.94	2.03	3.21

FOLD4	client A	1.06	1.06	0.82	1.24	1.04
	client B	1.09	1.19	0.81	1.48	1.14
	client C	0.78	2.05	1.48	0.99	1.33
	client D	0.8	2.26	1.58	0.93	1.39
FOLD5	client A	1.69	1.59	2.47	1.57	1.83
	client B	1.77	2.02	2.52	1.64	1.99
	client C	0.92	2.05	5.39	1.55	2.48
	client D	0.93	2.13	5.51	1.48	2.51

5. FedRep

T1-DUAL DSC

		Liver	Right Kidney	Left Kidney	Spleen	Mean
FOLD1	client A	65.76	43.79	49.28	73.91	58.18
	client B	67.49	41.78	47.08	75.21	57.89
	client C	65.08	43.51	48.88	74.51	57.99
	client D	65.68	43.61	48.83	72.07	57.55
FOLD2	client A	78.87	50.62	42.6	66.83	59.73
	client B	74.34	48.32	41.23	66.69	57.64
	client C	74.07	48.84	39.4	64.83	56.78
	client D	74.79	48.62	38.86	64.71	56.75
FOLD3	client A	80.62	52.36	58.35	69.45	65.19
	client B	78.23	45.99	54.14	59.51	59.47
	client C	77.88	48.61	55.52	64.18	61.55
	client D	77.08	48.48	55.64	61.52	60.68
FOLD4	client A	82.53	57.11	48.52	72.04	65.05
	client B	83	56.93	49.76	73.11	65.7
	client C	81.85	53.46	47.74	72.38	63.86
	client D	82.75	54.52	50.23	74.28	65.44
FOLD5	client A	71.1	47.78	59.34	64.6	60.7
	client B	70.43	44.8	58.31	67.26	60.2
	client C	66.81	47.73	55.31	63.67	58.38
	client D	65.46	49.27	53.97	63.41	58.03

T1-DUAL ASSD

		Liver	Right Kidney	Left Kidney	Spleen	Mean
FOLD1	client A	2.72	2.71	2.04	2.59	2.51
	client B	2.73	2.65	1.88	2.77	2.51
	client C	3.09	3.1	2.64	2.8	2.91
	client D	3.13	3.01	3.13	2.98	3.06
FOLD2	client A	2.8	2.77	3.59	2.9	3.01
	client B	2.77	2.75	3.43	3	2.99

	client C	3.13	2.68	3.34	2.93	3.02
	client D	3.01	2.69	3.29	2.95	2.98
FOLD3	client A	0.96	1.94	0.97	2.18	1.51
	client B	0.95	2.07	0.95	1.94	1.48
	client C	0.74	2.21	0.96	1.95	1.47
	client D	0.78	2.33	0.99	2.13	1.55
FOLD4	client A	0.85	3.08	4.41	1.04	2.34
	client B	0.82	3.09	4.3	1.08	2.32
	client C	0.65	2.65	5.08	0.86	2.31
	client D	0.64	2.65	4.76	0.81	2.21
FOLD5	client A	1.37	1.29	0.78	2.49	1.48
	client B	1.41	1.34	0.8	2.48	1.51
	client C	1.36	1.32	0.99	2.25	1.48
	client D	1.38	1.35	0.96	2.15	1.46

T2-SPIR　DSC

		Liver	Right Kidney	Left Kidney	Spleen	Mean
FOLD1	client A	82.67	75.84	81.75	87.68	81.98
	client B	82.34	74.99	80.68	87.5	81.38
	client C	83.99	71.93	81.09	86.14	80.79
	client D	83.69	71.82	81.41	86.1	80.76
FOLD2	client A	83.67	82.18	70.14	85.01	80.25
	client B	83.53	80.83	70.08	84.89	79.83
	client C	84.18	76.26	67	84.31	77.94
	client D	84.01	76.03	67.66	84.33	78.01
FOLD3	client A	79.56	76.32	75.11	87.42	79.6
	client B	79.33	76.42	75.61	87.74	79.77
	client C	80.96	75.83	75.73	87.65	80.04
	client D	80.97	75.56	76.03	87.69	80.06
FOLD4	client A	76.85	79.19	79.68	85.36	80.27
	client B	76.8	79.33	79.57	85.42	80.28
	client C	78.86	77.63	78.88	83.86	79.81
	client D	78.65	77.87	78.57	83.45	79.63
FOLD5	client A	77.13	84.88	80.84	88.44	82.82
	client B	76.91	84.24	80.4	88.11	82.42
	client C	79.04	78.97	79.98	87.99	81.49
	client D	78.56	78.16	80.09	88.18	81.25

T2-SPIR　ASSD

		Liver	Right Kidney	Left Kidney	Spleen	Mean
FOLD1	client A	0.53	3.28	0.64	0.79	1.31

	client B	0.59	3.64	0.68	0.64	1.39
	client C	0.49	3.99	0.71	1.13	1.58
	client D	0.52	4.04	0.69	1.12	1.59
FOLD2	client A	0.93	1.16	1.58	1.09	1.19
	client B	0.97	1.33	1.74	1.13	1.3
	client C	0.81	1.91	2.75	1.24	1.68
	client D	0.79	1.89	2.72	1.3	1.67
FOLD3	client A	0.98	1.62	2.7	0.6	1.47
	client B	0.99	1.65	2.47	0.55	1.41
	client C	0.86	1.81	2.72	0.63	1.5
	client D	0.85	1.83	2.73	0.6	1.5
FOLD4	client A	1.36	2.02	0.88	1.38	1.41
	client B	1.35	2.15	0.86	1.31	1.42
	client C	0.92	2.73	1.27	1.7	1.65
	client D	0.92	2.77	1.28	1.75	1.68
FOLD5	client A	0.99	0.57	1.47	0.31	0.84
	client B	0.99	0.65	1.72	0.44	0.95
	client C	0.79	1.18	2.4	0.72	1.27
	client D	0.83	1.26	2.3	0.69	1.27

6. LG-FedAVG

T1-DUAL DSC

		Liver	Right Kidney	Left Kidney	Spleen	Mean
FOLD1	client A	50.7	28.34	24.52	42.17	36.43
	client B	48.08	21.17	35.07	37.44	35.44
	client C	48.98	22.04	24.73	35.58	32.83
	client D	48.81	24.43	26.51	34.94	33.67
FOLD2	client A	47.29	9.93	12.73	52.65	30.65
	client B	46.27	1.41	36.67	35.14	29.87
	client C	49.93	23.26	24.16	35.61	33.24
	client D	48.96	19.83	24.31	37.11	32.55
FOLD3	client A	49.16	5.34	19.02	55.39	32.23
	client B	48.05	7.34	26.94	45.06	31.85
	client C	56.79	26.31	23.75	30.86	34.43
	client D	54.08	25.37	25.84	41.02	36.58
FOLD4	client A	58.42	2.88	14.12	65.24	35.17
	client B	54.66	0.25	44.07	66.97	41.48
	client C	49.54	30.87	27	40.84	37.06
	client D	49.77	29.82	22.92	45.9	37.11
FOLD5	client A	52.04	23.67	1.75	51.41	32.22
	client B	59.22	3.42	25.34	48.1	34.02
	client C	57.77	24.48	30.07	38.37	37.67

	client D	52.35	24.09	29.78	38.33	36.14

T1-DUAL ASSD

		Liver	Right Kidney	Left Kidney	Spleen	Mean
FOLD1	client A	5.9	8.92	2.34	9.87	6.76
	client B	2.4	9.46	1.54	10.6	6
	client C	1.8	3.68	2.23	2	2.43
	client D	1.63	4.34	1.88	2.26	2.53
FOLD2	client A	2.63	9.31	2.93	1.79	4.16
	client B	2.22	10.2	4.04	4.96	5.36
	client C	2.07	3.12	2.26	2.95	2.6
	client D	2.15	4.38	2.08	2.6	2.8
FOLD3	client A	2.17	6.66	20.22	3.12	8.04
	client B	2.43	6.2	18.16	1.72	7.13
	client C	1.45	2.48	8.78	2.78	3.87
	client D	1.39	2.83	5.63	1.81	2.91
FOLD4	client A	1.35	6.06	3.07	0.74	2.8
	client B	1.35	9.64	2.15	1.04	3.55
	client C	1.83	1.74	4.3	1.72	2.4
	client D	1.69	2.44	5.61	1.97	2.93
FOLD5	client A	2.69	2.33	21.05	1.73	6.95
	client B	1.04	5.21	3.8	1.51	2.89
	client C	1.26	2.46	2.57	1.86	2.04
	client D	1.59	2.64	1.96	2.29	2.12

T2-SPIR DSC

		Liver	Right Kidney	Left Kidney	Spleen	Mean
FOLD1	client A	56.31	2.99	6.22	67.07	33.15
	client B	51.15	1.63	15.71	61.55	32.51
	client C	50.47	52.31	47.76	41.68	48.05
	client D	45.36	42.35	55.33	34.29	44.33
FOLD2	client A	55.08	0.66	9.64	65.47	32.71
	client B	52.1	0.05	24.42	64.7	35.32
	client C	48.88	48.07	55	48.39	50.09
	client D	47.3	45.79	48.45	31.92	43.37
FOLD3	client A	60.28	5.6	2.69	68.29	34.22
	client B	55	0.3	18.78	67.13	35.3
	client C	47.11	49.53	45.21	55.62	49.37
	client D	47.27	51.97	43.68	30.28	43.3
FOLD4	client A	63.25	0.46	23.52	58	36.31
	client B	62.51	1.2	13.87	63.95	35.38

	client C	57.63	47.08	40.21	49.45	48.59
	client D	53.26	47.69	27.19	28.55	39.17
FOLD5	client A	59.64	1.34	31.13	70.33	40.61
	client B	57.06	1.34	15.94	66.11	35.11
	client C	46.78	50.87	41.31	45.06	46.01
	client D	52	56.29	41.37	32.87	45.63

T2-SPIR ASSD

		Liver	Right Kidney	Left Kidney	Spleen	Mean
FOLD1	client A	1.61	18.87	3.92	1.28	6.42
	client B	2.93	26.89	3.73	3.26	9.2
	client C	1.81	4.87	3.21	8.55	4.61
	client D	2.54	8.12	2.29	6.37	4.83
FOLD2	client A	1.55	10.21	6.09	1.34	4.8
	client B	1.37	14.36	4.84	2.77	5.83
	client C	1.6	5.57	7.3	5.74	5.05
	client D	1.92	10.63	7.19	5.3	6.26
FOLD3	client A	1.14	9.29	6.03	1.07	4.38
	client B	1.35	10.89	4.6	1.71	4.64
	client C	2.14	7.2	6.07	2.3	4.43
	client D	2.66	5.67	4.53	5.15	4.5
FOLD4	client A	1.1	18.55	15.18	4.3	9.78
	client B	0.96	8.74	9.62	4.08	5.85
	client C	1.2	8.5	9.39	4.55	5.91
	client D	0.97	5.25	3.9	1.31	2.86
FOLD5	client A	1.5	6.35	7.55	2.43	4.46
	client B	2.44	5.95	5.33	3.76	4.37
	client C	2.12	4.77	7.28	3.97	4.53
	client D	1.61	18.87	3.92	1.28	6.42

7. Fedprox

T1-DUAL DSC

		Liver	Right Kidney	Left Kidney	Spleen	Mean
FOLD1	client A	61.09	42.93	47.04	68.23	54.8225
	client B	59.95	42.58	48.62	70.62	55.4425
	client C	58.38	42.63	47.22	69.35	54.395
	client D	59.21	43.87	47.81	68.06	54.7375
FOLD2	client A	71.29	46.26	56.78	64.54	59.7175
	client B	71.28	47.47	57.92	64.23	60.225
	client C	72.41	47.32	56.14	64.85	60.18
	client D	70.43	45.44	57.93	64.02	59.455

FOLD3	client A	82.25	64.67	61.14	76.92	71.245
	client B	82.15	64.03	61.29	72.54	70.0025
	client C	80.23	63.17	61.15	72.18	69.1825
	client D	80.97	60.64	60.55	73.57	68.9325
FOLD4	client A	82.27	50.63	61.56	72.28	66.685
	client B	81.49	50.62	59.01	73.26	66.095
	client C	81.62	49.78	58.05	73.23	65.67
	client D	83.63	48.4	58.16	73.57	65.94
FOLD5	client A	69.46	56.21	59.29	58.6	60.89
	client B	68.45	53.21	58.95	60.53	60.285
	client C	66.48	51.52	59.15	58.4	58.8875
	client D	67.93	53.44	59.12	58.09	59.645

T1-DUAL ASSD

		Liver	Right Kidney	Left Kidney	Spleen	Mean
FOLD1	client A	1.3	4.61	5.36	4.22	3.87
	client B	1.51	9.85	4.27	1.4	4.26
	client C	1.5	7.3	3.05	1.18	3.26
	client D	1.58	6.58	3.09	0.98	3.06
FOLD2	client A	1.05	4.23	5.66	3.53	3.62
	client B	1.05	4.23	5.66	3.53	3.62
	client C	1.44	5.98	5.43	4.21	4.26
	client D	1.3	4.61	5.36	4.22	3.87
FOLD3	client A	0.67	3.38	1.1	1.83	1.74
	client B	0.78	4.47	1.71	1.92	2.22
	client C	0.76	4.3	1.63	1.88	2.14
	client D	0.88	4.8	1.83	2.5	2.5
FOLD4	client A	0.56	2.92	4.42	1.56	2.36
	client B	0.53	3.08	4.08	1.43	2.28
	client C	0.51	3.6	4.33	1.23	2.41
	client D	0.51	3.6	4.33	1.23	2.41
FOLD5	client A	1.88	2.46	1.13	1.32	1.7
	client B	2.59	2.37	1.36	1.13	1.86
	client C	2.82	2.02	1.19	1.2	1.81
	client D	1.74	2.97	1.38	1.47	1.89

T2-SPIR DSC

		Liver	Right Kidney	Left Kidney	Spleen	Mean
FOLD1	client A	83.33	82.08	77.8	82.43	81.41
	client B	83.33	82.08	77.8	82.43	81.41
	client C	83.33	82.08	77.8	82.43	81.41

	client D	83.33	82.08	77.8	82.43	81.41
FOLD2	client A	82.85	78.5	64.16	83.16	77.17
	client B	82.36	76.66	66.56	84.45	77.51
	client C	80.8	74.05	61.54	82.35	74.69
	client D	82.92	76.54	61.8	81.57	75.71
FOLD3	client A	81.27	77.82	73.58	86.36	79.76
	client B	81.45	76	71.75	83.87	78.27
	client C	81.5	76.66	69.91	83.02	77.77
	client D	79.09	77.06	71.85	82.8	77.7
FOLD4	client A	80.55	74.86	80.82	82.07	79.57
	client B	75.58	76.47	81.18	79.71	78.24
	client C	76.8	75.74	80.57	80.49	78.4
	client D	76.8	75.74	80.57	80.49	78.4
FOLD5	client A	78.08	83.7	79.04	87.2	82.01
	client B	78.11	82.42	79.68	85.84	81.51
	client C	74.5	81.87	76.86	84.96	79.55
	client D	74.49	81.8	75.35	83.2	78.71

T2-SPIR ASSD

		Liver	Right Kidney	Left Kidney	Spleen	Mean
FOLD1	client A	1.58	6.58	3.09	0.98	3.06
	client B	0.47	1.2	1.52	3.48	1.67
	client C	0.5	1.41	0.71	1.75	1.09
	client D	0.48	1.09	1	2.8	1.34
FOLD2	client A	0.48	1.65	3.33	1.36	1.7
	client B	0.48	1.8	3.44	1.5	1.81
	client C	0.48	1.8	3.44	1.5	1.81
	client D	0.57	1.78	3.01	1.56	1.73
FOLD3	client A	0.59	1.62	2.55	0.57	1.33
	client B	0.65	1.42	1.87	0.56	1.13
	client C	0.54	1.46	1.97	0.63	1.15
	client D	0.61	1.43	2.38	0.81	1.31
FOLD4	client A	0.82	3.37	0.51	2.24	1.74
	client B	0.82	3.07	0.64	2.2	1.68
	client C	1.12	1.87	0.5	2.29	1.44
	client D	1.33	2.06	0.6	1.93	1.48
FOLD5	client A	0.81	0.81	0.54	0.3	0.61
	client B	1.07	0.62	0.48	0.5	0.67
	client C	1.22	0.49	0.6	0.5	0.7
	client D	1.22	0.49	0.6	0.5	0.7

8.PAF-Fed (Ours)

T1-DUAL DSC

		Liver	Right Kidney	Left Kidney	Spleen	Mean
FOLD1	client A	70.22	44.34	57.83	73.19	61.4
	client B	70.72	44.08	58	73.12	61.48
	client C	73.65	42.94	56.63	70.44	60.92
	client D	74	42.97	56.9	71.01	61.22
FOLD2	client A	73.25	44.81	49.65	69.08	59.2
	client B	73.78	45.25	49.55	68.99	59.39
	client C	75.89	44.13	49.09	67.51	59.15
	client D	76.47	43.7	48.5	67.53	59.05
FOLD3	client A	81.12	54.91	61.29	72.4	67.43
	client B	81.54	54.65	60.9	72.37	67.36
	client C	84.37	53.59	60.85	70.27	67.27
	client D	84.7	53.74	60.75	70.01	67.3
FOLD4	client A	81.2	53.45	58.96	73.37	66.74
	client B	81.56	52.83	59.05	72.59	66.51
	client C	83.56	50.49	59.01	70.51	65.89
	client D	83.54	50.38	59.33	70.53	65.95
FOLD5	client A	77.9	53.8	60.37	69.68	65.44
	client B	78.75	54.36	60.87	69.72	65.92
	client C	81.88	51.75	59.77	68.02	65.36
	client D	81.96	51.73	58.93	68.11	65.18

T1-DUAL ASSD

		Liver	Right Kidney	Left Kidney	Spleen	Mean
FOLD1	client A	0.98	1.31	0.99	0.95	1.06
	client B	0.94	1.3	1.02	0.95	1.06
	client C	0.72	1.96	1.99	1	1.42
	client D	0.71	1.94	1.88	0.97	1.38
FOLD2	client A	1.68	2.06	1.4	1.36	1.62
	client B	1.3	1.88	1.42	1.38	1.5
	client C	0.9	1.89	1.19	1.43	1.35
	client D	0.85	1.88	1.19	1.45	1.34
FOLD3	client A	2.27	2.46	1.34	2.41	2.12
	client B	2.08	2.26	1.32	2.33	2
	client C	1.27	2.45	4.16	2.9	2.7
	client D	1.28	2.49	4.04	2.65	2.61
FOLD4	client A	0.67	3.97	0.96	1.18	1.7
	client B	0.65	4.12	0.95	1.18	1.73
	client C	0.57	4.95	1.18	1.15	1.96
	client D	0.57	5.07	1.18	1.17	2

FOLD5	client A	0.68	1.63	1.22	1.34	1.22
	client B	0.65	1.6	1.23	1.39	1.21
	client C	0.57	1.39	1.01	1.41	1.1
	client D	0.57	1.42	1.01	1.44	1.11

T2-SPIR DSC

		Liver	Right Kidney	Left Kidney	Spleen	Mean
FOLD1	client A	82.84	80.28	76.94	83.7	80.94
	client B	82.39	80.1	77.37	83.33	80.8
	client C	84.61	76.55	79.92	83.55	81.16
	client D	84.6	76.03	80.8	83.67	81.28
FOLD2	client A	80.57	84.37	71.69	87.17	80.95
	client B	80.62	84.19	71.21	87.09	80.78
	client C	81.62	82.22	69.74	85.95	79.88
	client D	81.74	82.29	69.98	85.93	79.98
FOLD3	client A	79.79	77.13	83.57	88.59	82.27
	client B	79.67	76.86	83.53	88.44	82.12
	client C	81.35	76.2	84.13	88.61	82.57
	client D	81.34	76.2	84.13	88.59	82.56
FOLD4	client A	79.85	78.93	74.3	86.21	79.82
	client B	79.74	79.98	74.33	86.25	80.08
	client C	82.02	78.83	72.55	84.97	79.59
	client D	81.83	78.91	73.23	85.01	79.74
FOLD5	client A	80.82	83.53	80.54	90.46	83.84
	client B	80.31	83.33	79.77	90.23	83.41
	client C	82.42	80.65	79.03	90.04	83.04
	client D	81.9	80.22	78.82	90	82.73

T2-SPIR ASSD

		Liver	Right Kidney	Left Kidney	Spleen	Mean
FOLD1	client A	0.41	1.99	0.93	1	1.08
	client B	0.38	1.42	1.25	1.24	1.07
	client C	0.4	1.71	1.05	1.54	1.18
	client D	0.41	2.01	1.01	1.32	1.19
FOLD2	client A	0.73	0.62	2.76	1.59	1.42
	client B	0.68	1.1	2.69	1.89	1.59
	client C	0.76	0.74	2.64	1.2	1.33
	client D	0.73	0.54	2.97	1.64	1.47
FOLD3	client A	0.58	2.36	1.19	0.77	1.22
	client B	0.63	1.88	1.08	1.01	1.15
	client C	0.54	1.78	1.14	0.89	1.08

	client D	0.57	2.59	1.14	1.04	1.34
FOLD4	client A	0.94	1.25	1.01	1.05	1.07
	client B	1.17	1.27	1.04	1.1	1.14
	client C	0.77	0.9	0.76	1.02	0.86
	client D	0.91	1.07	1.37	1.22	1.14
FOLD5	client A	0.78	1.16	0.86	0.48	0.82
	client B	0.74	1.31	0.62	0.43	0.77
	client C	0.77	1.13	0.91	0.46	0.82
	client D	0.79	1.09	0.73	0.62	0.81
